# NLRP3 inflammasome: Pathogenic role and potential therapeutic target for IgA nephropathy

**DOI:** 10.1038/srep41123

**Published:** 2017-01-24

**Authors:** Yu-Ling Tsai, Kuo-Feng Hua, Ann Chen, Chyou-Wei Wei, Wen-Shiang Chen, Cheng-Yeu Wu, Ching-Liang Chu, Yung-Luen Yu, Chia-Wen Lo, Shuk-Man Ka

**Affiliations:** 1 Graduate Institute of Life Sciences, National Defense Medical Center, Taipei, Taiwan, ROC; 2 Department of Biotechnology and Animal Science, National Ilan University, Ilan, Taiwan, ROC; 3 Department of Pathology, Tri-Service General Hospital, National Defense Medical Center, Taipei, Taiwan, ROC; 4 Department of Nutrition, Hungkuang University, Taichung, Taiwan, ROC; 5 Department of Physical Medicine and Rehabilitation, National Taiwan University Hospital and National Taiwan University College of Medicine, Taipei, Taiwan, ROC; 6 Molecular Genetics Laboratory, Department of Microbiology and Immunology, Chang-Gung University, Taoyuan, Taiwan, ROC; 7 Graduate Institute of Immunology, College of Medicine, National Taiwan University, Taipei, Taiwan, ROC; 8 Graduate Institute of Cancer Biology and Center for Molecular Medicine, China Medical University, Taichung, Taiwan, ROC; 9 Graduate Institute of Aerospace and Undersea Medicine, Academy of Medicine, National Defense Medical Center, Taipei, Taiwan, ROC

## Abstract

We have previously showed that IL-1β is involved in the pathogenesis of both spontaneously occurring and passively induced IgA nephropathy (IgAN) models. However, the exact causal-relationship between NLRP3 inflammasome and the pathogenesis of IgAN remains unknown. In the present study, we showed that [1] IgA immune complexes (ICs) activated NLRP3 inflammasome in macrophages involving disruption of mitochondrial integrity and induction of mitochondrial ROS, bone marrow-derived dendritic cells (BMDCs) and renal intrinsic cells; [2] knockout of NLRP3 inhibited IgA ICs-mediated activation of BMDCs and T cells; and [3] knockout of NLRP3 or a kidney-targeting delivery of shRNA of NLRP3 improved renal function and renal injury in a mouse IgAN model. These results strongly suggest that NLRP3 inflammasome serves as a key player in the pathogenesis of IgAN partly through activation of T cells and mitochondrial ROS production and that a local, kidney-targeting suppression of NLRP3 be a therapeutic strategy for IgAN.

IgA nephropathy (IgAN) is the most common primary glomerulonephritis and characterized by various degrees of intrinsic cell proliferation, especially mesangial cells (MCs), in the affected glomerulus, and mononuclear leukocyte infiltration in the glomerulus and renal interstitium^1^,^2^,^3^,^4^, with insidious progression to end-stage renal disease in up to 50% of the patients^5^,^6^. Deposition of predominantly glomerular IgA immune complexes (ICs) or IgA immune aggregates^1^,^6^,^7^, and activation of innate immunity, followed by T cell activation and resultant inflammatory responses have been considered as a leading cause of the disease^3^,^7^,^8^. However, the exact pathogenic mechanism underlying IgAN remains largely unknown, and specific treatment for the renal disease is still insufficient. NLRP3 inflammasome links innate and adaptive immunity and is involved in secretion of IL-1β and IL-18 by various immune cells^9^,^10^,^11^,^12^ in an inflammatory condition^13^,^14^,^15^,^16^. Innate immunity is triggered by endogenous or environmental insults through assembly of the NLRP3 inflammasome^10^,^12^, which has been observed in many cell types, such as macrophages, dendritic cells (DCs), T cells, and some epithelial cells^10^,^11^,^12^. Recently, several studies have suggested that NLRP3 inflammasome may be implicated in the pathogenesis of IgAN in pateints^16^,^17^,^18^. However, the causal-relationship between the inflammasome and the pathogenesis of IgAN is yet to be determined.

We have previously showed that [1] IL-1β and related molecular mechanisms are involved in the pathogenesis of a spontaneously occurring IgAN model in ddY mice^19^, and [2] protein levels of NLRP3 and IL-1β, and caspase-1 activity in the kidney are significantly increased in a passively induced mouse IgAN model, but this effect is inhibited by potent chemical inhibitors that are originally derived from traditional Chinese medicines^20^,^21^, suggesting that NLRP3 inflammasome may play a role in the pathogenesis of IgAN.

Herein, we hypothesized that activation of NLRP3 inflammasome contributes to the pathogenesis of IgAN and targeting the inflammasome may be beneficial for the treatment of the renal disease. This is the first report that addresses the NLRP3 inflammasome activation specifically involved in the development and progression of IgAN, based on our mechanistic investigations using [1] cultured macrophages, DCs, glomerular MCs, and renal tubular epithelial cells (TECs), [2] NLRP3 knockout (NLRP3 KO) mice, and [3] gene delivery of shRNA using a kidney-targeting, ultrasound-mediated microbubble technique.

## Results

### IgA ICs induces IL-1β secretion through NLRP3 inflammasome in macrophages and DCs, and subsequent T cells activation

**Figure 1:**
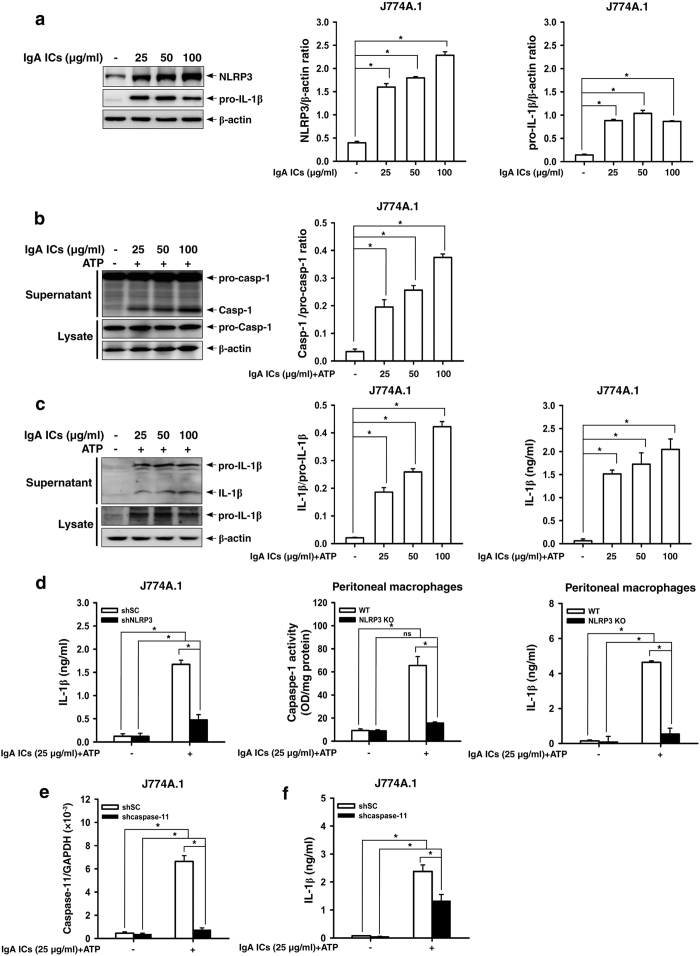
NLRP3 inflammasome activation in IgA ICs-primed macrophages. NLRP3 and pro-IL-1β levels by Western blotting after incubated for 6 h with IgA ICs and semi-quantitative analysis in J774A.1 macrophages **(a)**. Caspase-1 levels by Western blotting and semi-quantitative analysis **(b)**, and IL-1β levels by Western blotting and semi-quantitative analysis and ELISA **(c)** in J774A.1 macrophages were incubated for 5.5 h with IgA ICs and 30 min ATP. IL-1β secretion or caspase-1 activity measured by ELISA in shSC or shNLRP3 J774A.1 macrophages and/or primary peritoneal macrophages from untreated wild type or NLRP3 KO mice, which were incubated for 5.5 h with IgA ICs and 30 min ATP **(d)**. Caspase-11 mRNA levels in cell lysate measured by real-time PCR **(e)** and IL-1β secretion by ELISA **(f)** in shSC or shcaspase-11 J774A.1 macrophages incubated for 5.5 h with IgA ICs and 30 min ATP. The data are expressed as the mean ± SEM for three separate experiments. *p < 0.05. WT, wild type. IgA ICs, IgA immune complexes. ns, no significant difference.

First, we examined whether IgA ICs can activate NLRP3 inflammasome in macrophages and DCs. As shown in Fig. 1, IgA ICs increased the protein levels of NLRP3 and pro-IL-1β in J774A.1 macrophages (Fig. 1a). IgA ICs induced caspase-1 activation in the presence of ATP evidenced by the increased active caspase-1 in the supernatants of J774A.1 cells (Fig. 1b). Also, IgA ICs induced IL-1β secretion in the presence of ATP as evidenced by increased levels of mature IL-1β in the supernatants detected by Western blot analysis and ELISA (Fig. 1c). As shown in Fig. 1d, IgA ICs induced IL-1β secretion was dependent on NLRP3 inflammasome, as the level of IL-1β secretion was significantly reduced in shRNA targeting NLRP3 macrophages (Fig. 1d). The role of NLRP3 in IgA ICs-induced IL-1β secretion was confirmed in peritoneal macrophages, as caspase-1 activation and IL-1β secretion were significantly reduced in the cells derived from NLRP3 KO mice compared to those of peritoneal macrophages from WT mice (Fig. 1d). In addition, a non-canonical pathway is implicated in the activation of the NLRP3 inflammasome, which, in mice, requires caspase-11 (while caspase-4 and caspase-5 in humans)^22^,^23^,^24^. As shown in Fig. 1e, IgA ICs activated shSC (shscramble) macrophages showed significantly higher caspase-11 mRNA levels than saline control, and these effects were either markedly inhibited or absent in J774A.1 macrophages stable transfection with shRNA targeting shcaspase-11. Furthermore, the levels of IL-1β were significantly lower in the shcaspase-11 cells than in the shSC cells (Fig. 1f).

**Figure 2:**
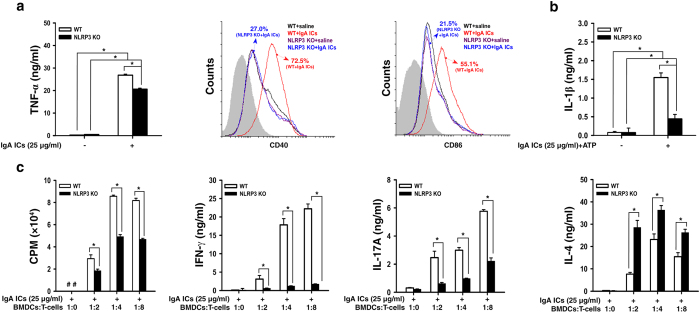
NLRP3 inflammasome activation in IgA ICs-primed dendritic cells. TNF-α secretion measured by ELISA, expression levels of CD40^+^ and CD86^+^ (within gated CD11c^+^ cells; the gray-filled area created by staining with an isotype-matched control antibody) determined by flow cytometry in BMDCs from untreated wild type or NLRP3 KO mice, which were incubated for 24 h with IgA ICs **(a)**. IL-1β secretion measured by ELISA in BMDCs from untreated wild type or NLRP3 KO mice, which were incubated for 24 h with IgA ICs and 30 min ATP **(b)**. T cell proliferation was measured by [3 H]-thymidine, and secretion of IFN-γ, IL-17A and IL-4 by ELISA at the indicated ratio of BMDCs:T cells for 3 days, in which BMDCs were incubated for 24 h with IgA ICs and cocultured with OT-II CD4^+^ T cell pulsed with OVA peptide **(c)**. The data are expressed as the mean ± SEM for three separate experiments. *p < 0.05. WT, wild type. #Not detectable. BMDCs, bone marrow derived dendritic cells. IgA ICs, IgA immune complexes.

**Figure 3:**
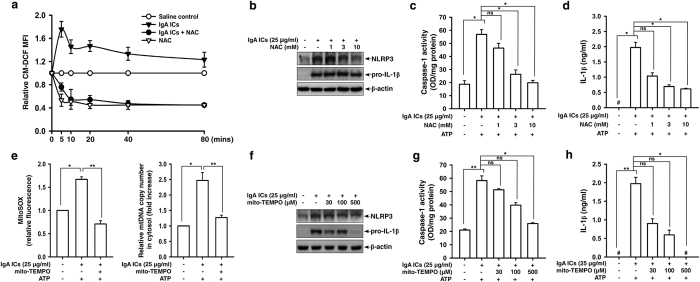
ROS production and mitochondrial homeostasis in IgA ICs-primed macrophages. ROS levels measured by 2′, 7′-dichlorofluorescein diacetate after incubated for 0–80 min with IgA ICs **(a)**. NLRP3 and pro-IL-1β levels by Western blotting after incubated for 6 h with IgA ICs **(b)**, caspase-1 activation **(c)** and IL-1β secretion **(d)** by ELISA after incubated for 5.5 h with IgA ICs and 30 min ATP in J774A.1 macrophages pre-incubated for 30 min with or without NAC. Mitochondrial ROS levels by MitoSOX after incubated for 5 min with IgA ICs and 30 min ATP, and mitochondrial DNA release into the cytosol by real-time RCR after incubated for 5.5 h with IgA ICs and 30 min ATP in J774A.1 macrophages pre-incubated for 30 min with or without mito-TEMPO **(e)**. NLRP3 and pro-IL-1β levels were analyzed by Western blotting after incubated for 6 h with IgA ICs **(f)**, and IL-1β secretion by ELISA after incubated for 5.5 h with IgA ICs and 30 min ATP **(g)** in J774A.1 macrophages pre-incubated for 30 min with or without mito-TEMPO. The data are expressed as the mean ± SEM for three separate experiments. *p < 0.05; **p < 0.001. #Not detectable. IgA ICs, IgA immune complexes. NAC, N-acetyl-L-cysteine. ns, no significant difference.

In addition, IgA ICs increased [1] TNF-α secretion, [2] percentages of CD11c^+^CD40^+^ and CD11c^+^CD86^+^ (Fig. 2a), and [3] IL-1β secretion (Fig. 2b) in bone marrow derived DCs (BMDCs) from WT mice, but this effect was significantly inhibited in BMDCs from NLRP3 KO mice. Using an OVA-specific T cell proliferation assay, IgA ICs-primed BMDCs from WT mice induced proliferation of CD4^+^ T cells and their secretion of IFN-γ, IL-17A and IL-4 (Fig. 2c), and, except IL-4, these effects were greatly inhibited in BMDCs from NLRP3 KO mice. These results suggest that NLRP3 inflammasome plays an important role in IgA ICs-mediated IL-1β secretion and T cell activation.

### IgA ICs induces NLRP3 inflammasome activation through reactive oxygen species (ROS) in macrophages

ROS is a key regulator of NLRP3 inflammasome^11^,^25^. We then investigated whether IgA ICs induced NLRP3 inflammasome activation through ROS-mediated pathways. We showed that IgA ICs induced ROS generation, but this effect was inhibited by N-acetyl-L-cysteine (NAC), an ROS scavenger (Fig. 3a). NAC also significantly reduced protein expression levels of NLRP3 and pro-IL-1β (Fig. 3b), caspase-1 activation (Fig. 3c) and IL-1β secretion (Fig. 3d) in IgA ICs-activated macrophages.

Mitochondrial ROS and oxidized mitochondrial DNA have been shown to play a crucial role in the process of NLRP3 inflammasome activation^12^,^26^. We showed that IgA ICs induced mitochondrial ROS generation and mitochondrial DNA release into the cytosol (Fig. 3e) of J774A.1 macrophages. In experiments using mito-TEMPO, an inhibitor of mitochondrial ROS, we showed that protein expression levels of NLRP3 and pro-IL-1β (Fig. 3f), caspase-1 activation (Fig. 3g), and IL-1β secretion (Fig. 3h) in IgA ICs-activated macrophages were significantly inhibited by mito-TEMPO.

### IgA ICs induces IL-1β secretion through NLRP3 inflammasome in renal intrinsic cells

**Figure 4:**
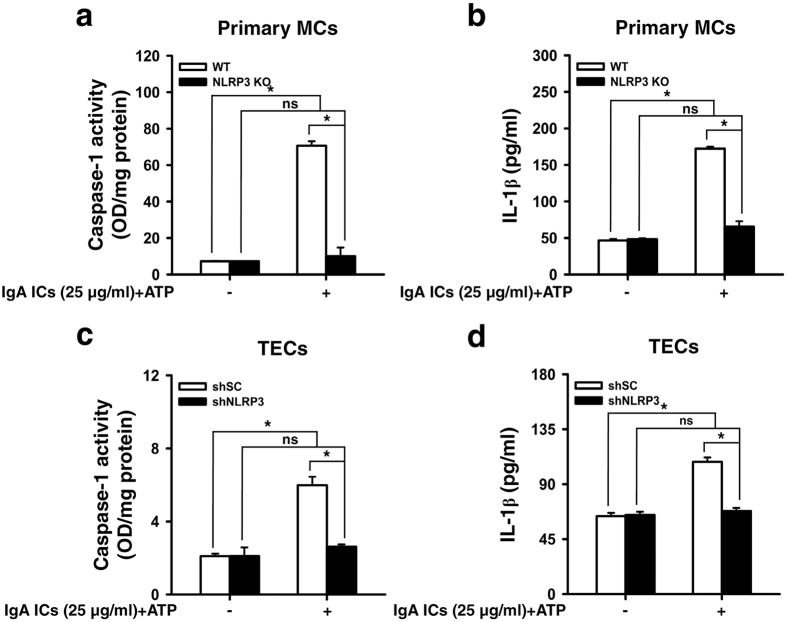
NLRP3 inflammasome activation in IgA ICs-primed renal intrinsic cells. Caspase-1 activity **(a)** and IL-1β secretion **(b)** measured by ELISA in glomerular primary MCs from untreated wild type or NLRP3 KO mice, which were incubated for 24 h with IgA ICs and 30 min ATP. Caspase-1 activity **(c)** and IL-1β secretion **(d)** measured by ELISA in renal TECs, which were incubated for 24 h with IgA ICs and 30 min ATP. The data are expressed as the mean ± SEM for three separate experiments. *p < 0.05. WT, wild type. ns, no significant difference. MCs, mesangial cells. TECs, tubular epithelial cells. IgA ICs, IgA immune complexes.

We tested whether IgA ICs can induce IL-1β secretion directly in renal intrinsic cells. The data show that IgA ICs induced caspase-1 activation (Fig. 4a) and IL-1β secretion (Fig. 4b) in primary MCs from WT mice, but these effects were significantly reduced in primary MCs from NLRP3 KO mice. IgA ICs also induced caspase-1 activation (Fig. 4c) and IL-1β secretion (Fig. 4d) in renal TECs transfection with shSC, but these effects were inhibited in TECs transfection with shNLRP3. These data suggest that NLRP3 inflammasome is involved in the IgA ICs-mediated inflammatory reaction in both the renal intrinsic cells.

### Improved proteinuria and renal function and milder renal lesions in IgAN in NLRP3 KO mice

**Figure 5:**
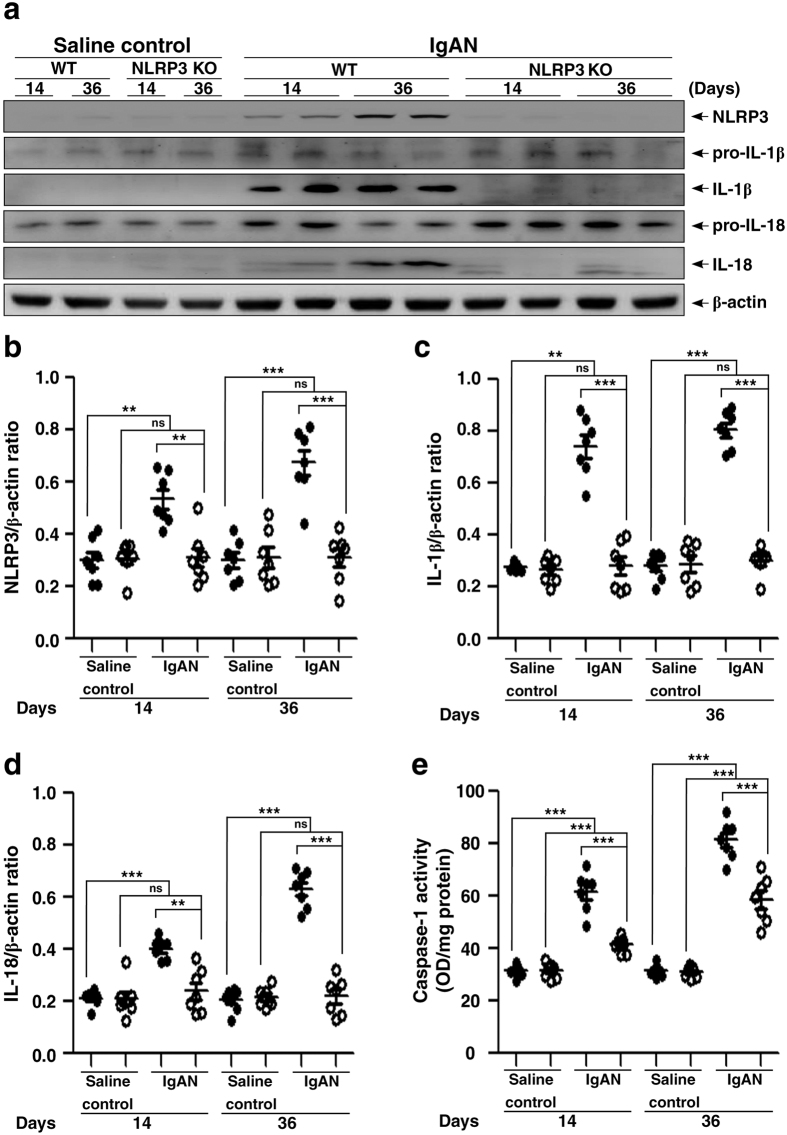
Renal NLRP3 inflammasome activation in wild type or NLRP3 KO mice with IgAN. Representative Western blots of NLRP3, IL-1β, or IL-18 levels in kidney tissues on day 14 and day 36, β-actin was used as the loading control **(a)**; semi-quantification of the NLRP3/β-actin ratio **(b)**, IL-1β/β-actin ratio **(c)**, or IL-18/β-actin ratio **(d).** Renal caspase 1 activity **(e)**. The data are expressed as the mean ± SEM for 7 mice per group. **p < 0.01; ***p < 0.005. WT, wild type. ns, no significant difference.

NLRP3 inflammasome controls caspase-1 activity and IL-1β release in various inflammatory diseases^13^,^14^,^15^,^16^, including IgAN^20^,^21^. We chose an IgAN model passively induced by repeated injections of IgA and pneumococcal C-polysaccharide (PnC) antigen to make it feasible inducing the experimental IgAN in NLRP3 KO mice (NLRP3 KO + IgAN mice), with which we examined the impact of lack of NLRP3 inflammasome activation on the development of the IgAN model. As shown in Fig. 5, compared to WT mice treated with saline (WT + saline mice), renal protein levels of NLRP3 (Fig. 5a,b), IL-1β (Fig. 5a,c) and IL-18 (Fig. 5a,d) were significantly increased in the IgAN model in WT mice (WT + IgAN mice) on days 14 and 36, but these effects were significantly inhibited in NLRP3 KO + IgAN mice. In addition, NLRP3 KO + IgAN mice showed much lower caspase-1 were observed in NLRP3 KO + IgAN mice, compared to NLRP3 KO mice treated with saline (NLRP3 KO + saline mice). There were no increased expression levels of renal NLRP3, IL-1β and IL-18 in NLRP3 KO + IgAN mice compared to NLRP3 KO + saline mice on days 14 and 36.

**Figure 6:**
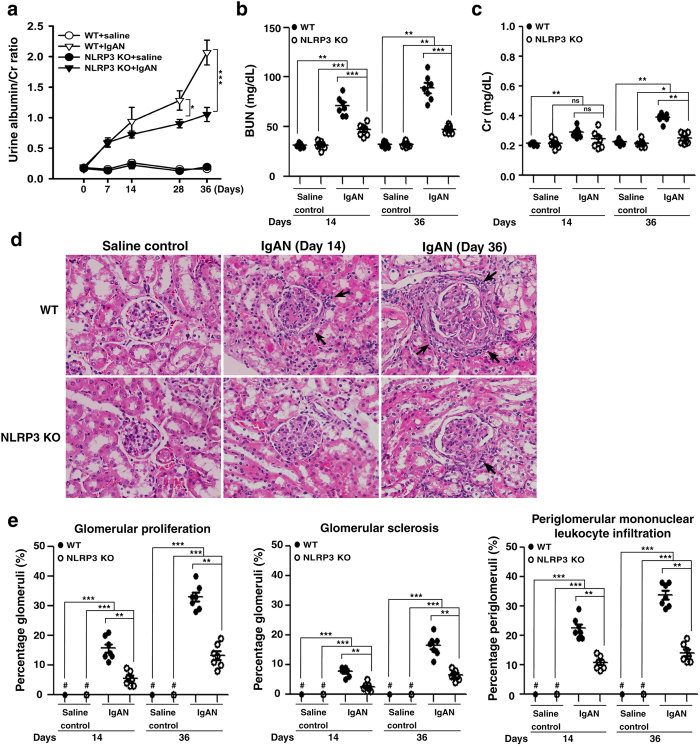
Albuminuria, renal function, and renal pathology in wild type or NLRP3 KO mice with IgAN. Time-course study of the urine albumin and Cr ratio **(a)**; BUN levels **(b)**; serum Cr levels **(c)**. Kidney histopathology (H&E staining) **(d)** on the indicated days, arrow indicates periglomerular mononuclear leukocyte infiltration, original magnification, 400x. Renal lesion scores **(e)**. The data are expressed as the mean ± SEM for 7 mice per group. *p < 0.05; **p < 0.01; ***p < 0.005. WT, wild type. #Not detectable. ns, no significant difference.

Besides, WT + IgAN mice presented increased albuminuria beginning on day 7 and persisting at high levels until day 36, as demonstrated by the urine albumin/creatinine (Cr) ratio, compared to WT + saline mice, whereas this effect was significantly decreased in NLRP3 KO + IgAN mice on days 28 and 36 (Fig. 6a). NLRP3 KO + IgAN mice still showed increased albuminuria compared to NLRP3 KO + saline mice.

In addition, compared to WT + saline mice, WT + IgAN mice showed significantly increased serum levels of blood urea nitrogen (BUN) (Fig. 6b) and Cr (Fig. 6c) on days 14 and 36, while, in NLRP3 KO + IgAN mice, BUN levels were significantly lower than in WT + IgAN mice on days 14 and 36 and Cr levels were significantly decreased on day 36. NLRP3 KO + IgAN mice showed increased serum levels of BUN and of Cr on days 14 and 36, compared to NLRP3 KO + saline mice.

Moreover, compared to WT + saline mice, WT + IgAN mice showed significantly increased levels of glomerular proliferation, glomerular sclerosis and periglomerular mononuclear leukocyte infiltration by light microscopy on days 14 and 36, and the severity of these renal lesions was greatly reduced in NLRP3 KO + IgAN mice (Fig. 6d,e). Increased levels of these renal lesions were observed in NLRP3 KO + IgAN mice on days 14 and 36 compared to NLRP3 KO + saline mice.

### Serum levels of IL-1β, TNF-α, IL-17A, IFN-γ, and IL-4 in IgAN in NLRP3 KO mice

The NLRP3 inflammasome plays a critical role in innate immunity and is involved in various inflammatory responses^9^,^10^,^12^ and Th1 and Th2 differentiation can be altered by activation of the NLRP3 inflammasome^11^,^14^,^27^,^28^. We therefore examined the effect of NLRP3 deficiency on systemic levels of proinflammatory cytokines in the mice. As shown in Table 1, on days 14 and 36, serum levels of IL-1β were significantly increased in WT + IgAN mice compared to WT + saline mice, but this effect was not seen in NLRP3 KO + IgAN mice. On days 14 and 36, serum levels of TNF-α were significantly increased in WT + IgAN mice compared to WT + saline mice and this effect was markedly lower in NLRP3 KO + IgAN mice on day 36. Serum levels of IL-17A showed no significant differences between the 4 groups on day 14, but a significantly increase was seen in WT + IgAN mice compared to WT + saline mice on day 36, which was not seen in the NLRP3 KO + IgAN mice. Serum levels of IL-4 showed no difference between the 4 groups on day 14, but a significant increase in the NLRP3 KO + IgAN mice compared to WT + IgAN mice on day 36. Increased serum levels of IFN-γ were seen in WT + IgAN mice on day 36, and this effect was lower in NLRP3 KO + IgAN mice on day 36, although there was no statistical significance between the mice and WT + IgAN mice. Besides, NLRP3 KO + IgAN mice showed increased serum levels of TNF-α (days 14 and 36) and IL-4 (day 36) compared to NLRP3 KO + saline mice. There was no significant difference in serum levels of IL-1β (days 14 and 36), IL-17A (days 14 and 36) and IL-4 (day 14) between NLRP3 KO + IgAN mice and NLRP3 KO + saline mice.

### Less infiltration of macrophages, DCs, and T cells into the kidney in NLRP3 KO + IgAN mice

**Figure 7:**
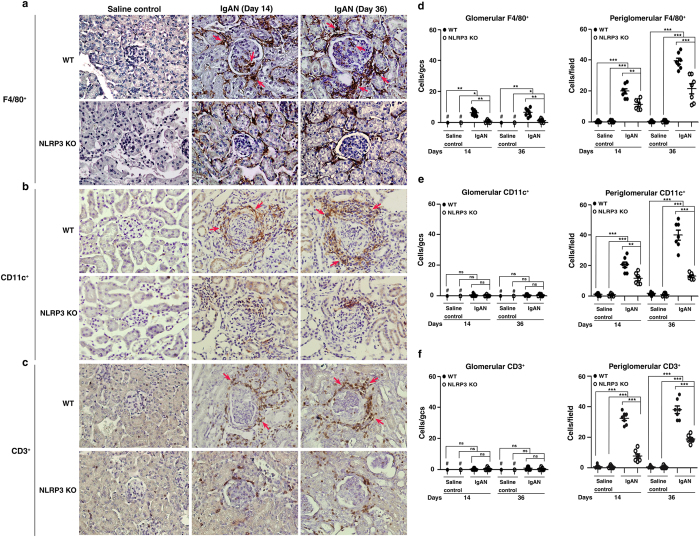
Infiltration of macrophages, DCs, or T cells of the kidney in wild type or NLRP3 KO mice with IgAN. F4/80^+^ macrophages **(a)**, CD11c^+^ DCs **(b)**, or CD3^+^ T cells **(c)** detected by immunohistochemistry, arrow indicates positive staining, original magnification, 400x; Scoring of stained cells in the glomerulus and periglomerular region **(d-f)**. The data are expressed as the mean ± SEM for 7 mice per group. *p < 0.05; **p < 0.01; ***p < 0.005. WT, wild type. #Not detectable. ns, no significant difference. gcs, glomerular cross section.

The NLRP3 inflammasome plays a crucial role in the innate immune response and links it to the adaptive immune response^8^,^10^,^11^,^27^,^28^. Notably, mononuclear leukocytes are often present around the inflamed glomeruli (periglomerular infiltration) in human IgAN^3^. In our study, on days 14 and 36, WT + IgAN mice showed markedly increased numbers of F4/80^+^ macrophages (both glomerular and periglomerular) (Fig. 7a,d), and CD11c^+^DCs (periglomerular) (Fig. 7b,e) and CD3^+^ T cells (periglomerular) (Fig. 7c,f) compared to WT + saline mice and these effects were significantly reduced in NLRP3 KO + IgAN mice. Increased numbers of periglomerular F4/80^+^ macrophages, CD11c^+^DCs and CD3^+^ T cells were observed in NLRP3 KO + IgAN mice on days 14 and 36, compared to NLRP3 KO + saline mice.

### Reduced T cells activation and increased T regulatory (Treg) cells in the spleen in NLRP3 KO + IgAN mice

**Figure 8:**
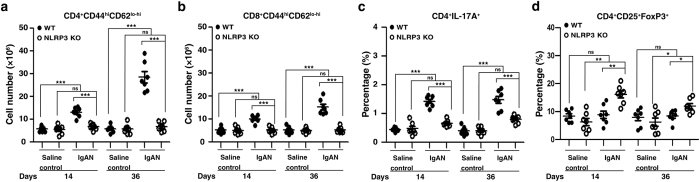
Memory T cells, CD4^+^ IL-17A^+^ T cells, and Treg cells of the spleen in wild type or NLRP3 KO mice with IgAN. Numbers of CD44^hi^ CD62L^lo-hi^ in CD4^+^ **(a)** or CD8^+^ T cells **(b)**. Percentage of CD4^+^ T cells expressing IL-17A^+^ **(c)**. Percentage of CD4^+^D25^+^ FoxP3^+^ Treg cells **(d)**. The data are expressed as the mean ± SEM for 7 mice per group. *p < 0.05; **p < 0.01; ***p < 0.005. WT, wild type. ns, no significant difference.

Deficiency of the NLRP3 inflammasome can attenuate systemic T cell immune responses in nephrotoxic serum nephritis^14^. We showed IgA ICs induced IL-1β secretion through NLRP3 inflammasome in macrophages and DCs, and subsequent T cells activation (Figs 1 and 2). These data suggest a role of the NLRP3 inflammasome in the interaction between these antigen presenting cells and T cells in IgAN. In the spleen, as shown in Fig. 8, WT + IgAN mice had significantly increased numbers of CD4^+^CD44^hi^CD62^lo-hi^ (Fig. 8a) and CD8^+^CD44^hi^CD62^lo-hi^ (Fig. 8b) effector/memory T cells compared to WT + saline mice on both days 14 and 36, and this effect was absent in NLRP3 KO + IgAN mice. In addition, the NLRP3 inflammasome mediates the production of IL-1β, which can further promote the differentiation of Th17 cells^27^,^29^. In our study, WT + IgAN mice had a significantly increased percentage of CD4^+^ IL-17A^+^ T cells in the spleen compared to WT + saline mice on both day 14 and day 36, and this effect was absent on day 14 and significantly reduced on day 36 in NLRP3 KO + IgAN mice (Fig. 8c). In addition, NLRP3 KO + IgAN mice had significantly increased percentage of CD4^+^CD25^+^ FoxP3^+^ Treg cells compared to WT + IgAN mice on days 14 and 36. There was no significant difference between NLRP3 KO + saline and WT + saline mice (Fig. 8d).

### Kidney-targeting shNLRP3 alleviates IgAN in mice

**Figure 9:**
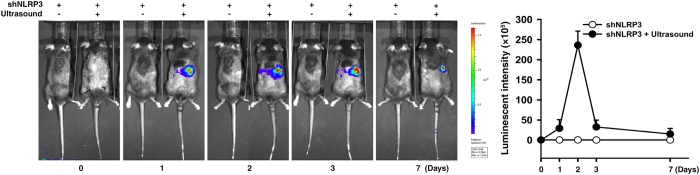
Determination of renal shNLRP3-luciferase activity by ultrasound-mediated microbubble gene delivery into mice. Luciferase activity in shNLRP3-luciferase mice measured each day after transfection with shNLRP3-luciferase by ultrasound-mediated microbubble gene delivery and an IVIS system. The ultrasound treatment was performed toward the right side of the back of the mice. The data are expressed as the mean ± SEM for 7 mice per group.

To further test whether blockade of NLRP3 has therapeutic potential for IgAN and thereby also confirmed the pathogenic role of NLRP3 inflammasome in the disease model, we used kidney-targeting, ultrasound-mediated microbubble gene delivery of shNLRP3 into the mice. First, we tested how long injected shNLRP3-luciferase survived *in vivo* and, as shown in Fig. 9, luciferase activity in the mice, as detected by an *in vivo* imaging system, peaked at day 2, although elevated expression was also seen at days 3 and 7. We therefore decided to inject the mice with shNLRP3 before and after induction of IgAN.

### Gene transfer before induction of IgAN

**Figure 10:**
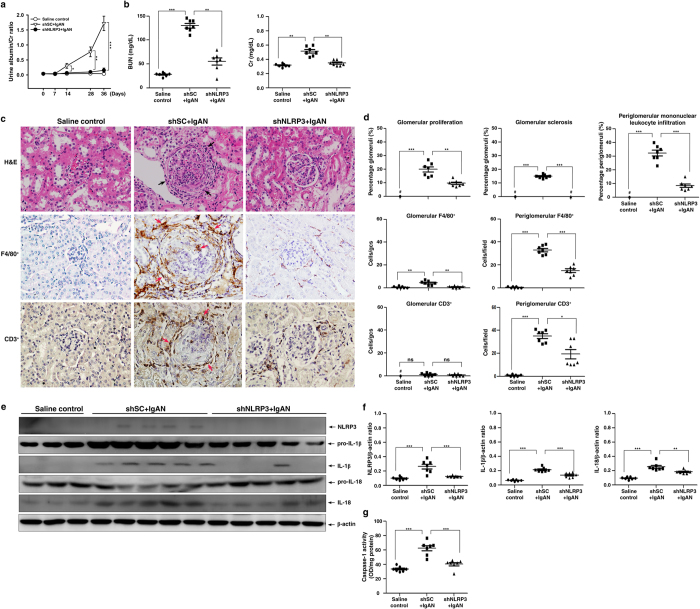
Kidney-targeting shNLRP3 delivery into mice before induction of IgAN. Urine albumin and Cr ratio on days 0, 7, 14, 28, and 36 **(a)**, serum levels of BUN and Cr on day 36 **(b)**. Kidney histopathology on day 36 [H&E staining] and F4/80^+^ macrophages and CD3^+^ T cells [IHC] **(c)**, original magnification, 400x; arrow indicates periglomerular mononuclear leukocyte infiltration or positive staining. Renal lesion scores and scoring of stained cells in the glomerulus and periglomerular region on day 36 **(d)**, NLRP3, IL-1β, and IL-18 protein levels on day 36 **(e)**, a typical result and the semiquantitative analysis for NLRP3, IL-1β, or IL-18 **(f)**, and renal caspase-1 activity **(g)**. The data are expressed as the mean ± SEM for 7 mice per group. *p < 0.05; **p < 0.01; ***p < 0.005. #Not detectable. ns, no significant difference. gcs, glomerular cross section.

shSC (shSC + IgAN) or shNLRP3 (shNLRP3 + IgAN) was injected every 3 days starting one day before induction of IgAN and tested the mice at various days. shNLRP3 + IgAN mice showed greatly reduced severity of disease compared to shSC + IgAN mice, including [1] significantly lower albuminuria levels on days 14 to 36 (Fig. 10a), [2] better renal function on day 36, as demonstrated by serum levels of BUN and Cr (Fig. 10b), [3] milder renal histopathology, such as lower glomerular proliferation, glomerular sclerosis, and periglomerular mononuclear leukocyte infiltration in the renal interstitium on day 36 (Fig. 10c,d), and [4] renal infiltration of macrophages (F4/80^+^) and T cells (CD3^+^) on day 36 (Fig. 10c,d).

In addition, although renal protein levels of NLRP3, IL-1β and IL-18 (Fig. 10e,f) and renal caspase-1 activity (Fig. 10g) on day 36 were increased in the shSC + IgAN mice compared to the controls, this effect was significantly inhibited in the shNLRP3 + IgAN mice.

### Gene transfer after induction of IgAN

**Figure 11:**
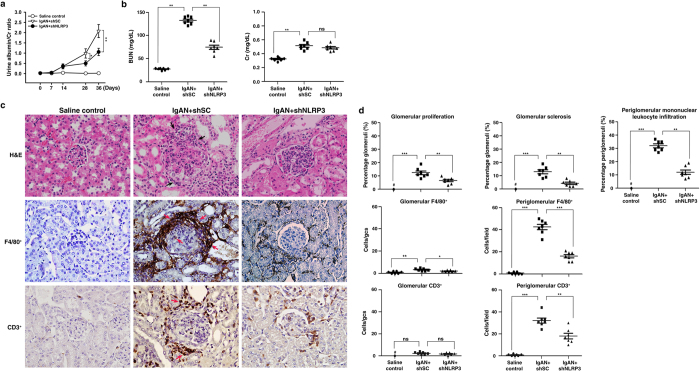
Kidney-targeting shNLRP3 delivery into mice after induction of IgAN. Urine albumin and Cr ratio on days 0, 7, 14, 28, and 36 **(a)**, serum levels of BUN and Cr on day 36 **(b)**. Kidney histopathology on day 36 [H&E staining] and F4/80^+^ macrophages and CD3^+^ T cells[IHC] **(c)**, original magnification, 400x; arrow indicates periglomerular mononuclear leukocyte infiltration or positive staining. Renal lesion scores and scoring of stained cells in the glomerulus and periglomerular region on day 36 **(d)**. The data show the mean ± SEM for results with 7 mice per group. *p < 0.05; **p < 0.01;***p < 0.005. #Not detectable. ns, no significant difference. gcs, glomerular cross section.

To mimic clinical status for treatment of IgAN, administration of shNLRP3 was started one week after the start of induction of IgAN in mice (IgAN + shNLRP3), which were sacrificed on day 36. Again, the results showed that IgAN + shNLRP3 mice demonstrated significantly improved renal conditions compared to IgAN + shSC mice, including [1] albuminuria levels (Fig. 11a); [2] renal function as demonstrated by serum levels of BUN, but not serum levels of Cr (Fig. 11b); [3] renal histopathology, such as glomerular proliferation, glomerular sclerosis, and periglomerular mononuclear leukocyte infiltration in the renal interstitium (Fig. 11c,d); and [4] renal infiltration of macrophages (F4/80^+^) and T cells (CD3^+^) (Fig. 11c,d).

The results from gene delivery with shRNA NLRP3 were consistent with the findings in the experiments involving the use of NLRP3 KO mice, suggesting that NLRP3 inflammasome activation plays a pathogenic role in IgAN and blockade of NLRP3 may have therapeutic potential for the disease.

## Discussion

The potential pathogenic role of NLRP3 inflammasome in IgAN remains unknown. In this study, we examined the causal-relationship between the NLRP3 inflammasome and the development of IgAN and identified the molecular mechanism underlying the pathogenic role of NLRP3 inflammasome in IgAN. Our data showed that [1] IgA ICs-induced NLRP3 inflammasome activation in macrophages involving disruption of mitochondrial integrity and induction of mitochondrial ROS, and renal intrinsic cells (e.g., MCs and renal TECs); [2] IgA ICs-induced DCs activation and resultant CD4^+^ T cells activation and differentiation/polarization; [3] improved renal function and histopathology in the IgAN model in NLRP3 KO mice or kidney-targeting gene delivery of shNLRP3. These findings suggest that the NLRP3 inflammasome plays a pathogenic role in the development of IgAN in part by activation of T cells and mitochondrial damage and ROS production. To our knowledge, this is the first report on the NLRP3 inflammasome specifically involved in both antigen-presenting cells and glomerular MCs and renal TECs in the development of an IgAN model in mice, although, in other glomerular and tubulointerstitial conditions, the inflammasome has been shown to be upregulated in immune cells, glomerular MCs, podocytes, or renal TECs^9^,^10^,^19^,^30^.

ROS released from mitochondria promotes mitochondrial permeability and facilitates the release of mitochondrial DNA into the cytosol, and the latter can activate the NLRP3 inflammasome^12^,^27^. We showed that IgA ICs induced mitochondrial dysfunction in macrophages, as evidenced by an increased mitochondrial ROS production and mitochondrial DNA copy numbers (Fig. 3e). The IgA ICs-induced increase in levels of NLRP3 and pro-IL-1β in (Fig. 3b,f) and IL-1β secretion (Fig. 3d,h) by macrophages was inhibited by two specific inhibitors of ROS, including NAC and mito-TEMPO. Together, the results suggest a central role of mitochondrial ROS in the IgA ICs-mediated activation of the inflammasome. These results suggest the importance of mitochondrial integrity in IgA ICs-induced NLRP3 inflammasome activation in macrophages. In addition to mitochondria, the roles of other cellular sources of ROS in NLRP3 inflammasome activation have been investigated. Recently, NADPH oxidase 4 (NOX4), a source of cellular superoxide anions, promotes NLRP3 inflammasome activation by increasing the expression of carnitine palmitoyltransferase 1A, a key mitochondrial enzyme in the fatty acid oxidation pathway^31^. Ives *et al*. showed that ROS derived by xanthine oxidase regulates NLRP3 activation in macrophages, leading to excessive IL-1β and IL-18 secretion^32^. In our previous studies, we demonstrated that ROS derived by cyclooxygenase-2 increases NLRP3 inflammasome activation by increasing LPS-induced NLRP3 expression and caspase-1 activation, and these effects were associated with increased NF-κB activation and mitochondrial damage, respectively^33^. Furthermore, ROS derived by NADPH oxidase regulates both NLRP3 expression and caspase-1 activation in macrophages^34^.

In addition, NLRP3 deficiency has been shown to inhibit systemic T cell immune responses in a mouse model of nephrotoxic serum nephritis through the activation of CD11c^+^ DCs^14^. In the present study, we showed that IgA ICs activated NLRP3 inflammasome in macrophages and DCs, and thereby activating T cells and in consistence with this, inhibited T cells activation in the spleen in NLRP3 KO + IgAN mice. Recently, we have shown that IL-17 is implicated in the pathogenesis of IgAN in the mouse model^20^, and it has been demonstrated that early Th17 cell differentiation involves IL-1 signaling in T cells^27^,^29^. In the present study, we showed that NLRP3 deficiency resulted in [1] decreased numbers of CD4^+^ IL-17A^+^ T cells (Fig. 8c) and increased percentage of Treg cells (Fig. 8d) in the mouse model of IgAN, and [2] reduced production of IL-17A and IFN-γ although enhanced production of IL-4, by activated T cells (Fig. 2c). An exaggerated pro-inflammatory T cell response in WT + IgAN mice, demonstrated by increased effector/memory T cells (Fig. 8a,b). This might be contributed an imbalance caused by a reduction in Treg cells (Fig. 8d), at least in part, by a restoration in Treg cells. In addition, it has been shown that reduced levels of IL-17A can inhibit Th1 responses^35^,^36^ and promote Th2 responses^38^ involving the activation of Treg cells. In consistence with these findings, in serum samples from NLRP3 KO + IgAN, levels of IL-17A and IFN-γ were decreased compared to those of WT + IgAN mice, although increased IL-4 levels were observed (Table 1).

In contrast, IL-4, which favors Th2 responses has a protective role in renal inflammatory responses^37^,^38^. Increased production of IL-4 in the CD4^+^ T cells activated by IgA ICs-primed BMDCs from NLRP3 KO mice, consistent with increased serum levels of the cytokine in the IgAN model of NLRP3 KO mice (the NLRP3 KO + IgAN mice), may further support the protective effect of NLRP3 deficiency in the IgAN mice. All together, our data support that the NLRP3 inflammasome plays a role in the T cell activation and differentiation/polarization underlying the observed renal inflammation in the mouse IgAN model.

On the other hand, our data also showed that a caspase-11 dependent, non-canonical pathway of NLRP3 inflammasome activation is involved in the IgA ICs-mediated activation of macrophages (Fig. 1e,f). In the caspase-11-mediated activation of noncanonical NLRP3 inflammasome, gram-negative bacterial LPS are sensed by TLR4, and LPS-mediated endocytosis of TLR4 induces IFN-α/β expression via activating IRF3-IRF7 complex. Secreted IFN-α/β binds to the IFNAR1/IFNAR2 receptor increasing the caspase-11 gene expression through JAK/STAT pathway^39^. Although the PnC antigen contained in the IgA ICs was prepared from Gram-positive *Streptococcus pneumoniae*, we found that IgA ICs increased caspase-11 mRNA levels in macrophages, this result suggested that IgA ICs might induce IFN-α/β expression. There are two possible mechanisms of caspase-11 activation. One is that the induction of procaspase-11 expression is necessary and sufficient for its auto-activation^40^. The other might be a mechanism mediated by unidentified scaffold/receptor that is induced by intracellular bacteria^40^. In the present study, we show that knockdown of caspase-11 inhibited IL-1β secretion induced by IgA ICs (Fig. 1f). This finding suggests that caspase-11 induced by IgA ICs might lead to auto-activation of caspase-11 and subsequence of caspase-1 activation and IL-1β secretion. However, we can’t exclude the possibility of uptake of IgA ICs by macrophages, thereby activating the caspase-11 as a similar finding observed in bacteria-mediated caspase-11 activation. Consistently, Andersen *et al*.^14^ reported that a non-canonical mechanism of NLRP3 inflammasome activation is involved in renal injury in nephrotoxic serum nephritis in mice. In the present study, however, whether a non-canonical pathway of NLRP3 inflammasome was operated in renal intrinsic cells (e.g., epithelial cells, MCs, and endothelial cells) in IgAN remains further investigation.

Very recently, Chun *et al*.^18^ reported that decreased expression of NLRP3 mRNA and protein in kidney biopsies of IgAN patients. The authors also showed that NLRP3 has been implicated in the pathogenesis of chronic kidney disease^16^. In view of their important findings and based on our data, we infer that the pathogenic role NLRP3 plays very likely in the initial or accelerating/progressing stages of the renal disorder, although further investigations are required to validate the hypothesis.

It should be noted that the mouse model of IgAN was significantly attenuated by injection of a kidney-targeting shNLRP3 before or after induction of the mouse IgAN model (Figs 10 and 11). Recently, two anti-NLRP3 compounds, MCC950^15^ and β-hydroxybutyrate^41^, were shown to reduce the severity of NLRP3 inflammasome-mediated experimental autoimmune encephalomyelitis, Muckle-Wells syndrome, and autoinflammatory syndrome, etc. These findings further support that administration of a kidney-targeting shNLRP3 may be a potential therapeutic for IgAN.

In this study, we decided to use the IgAN model passively induced by repeated injections of IgA and PnC antigen so that it was more feasible in performing the induction of the experimental IgAN in NLRP3 KO mice, and also because it was indeed a very stable IgAN model to reproduce the characteristic granular immunofluorescence pattern of IgA and C3 mesangial deposits, with renal inflammation and fibrosis^7^,^20^,^21^,^42^,^43^ and has been used in various experiments involving [1] the role of different IgA subtypes in IgA ICs glomerular deposition, [2] complement activation and ICs deposition, [3] the clearance kinetics of circulating IgA ICs and the role of hepatic Kupffer cells in their elimination, [4] the impact of the nature of antigen in IgA ICs in resultant renal injury, and [5] the synergy between extra-renal cytokines and IgA mesangial deposits in the development of renal injury and dysfunction and in the evolution of renal histopathologic changes.

However, in the present study, the exact role of the inflammasome in antigen-presenting cells and glomerular cells is not clearly established, this would require cell-specific gene-inactivation or at least bone marrow transplantation^44^. In addition, we could not exclude the possibility of other inflammasomes^45^ or their related pathways^46^ also involved in the mouse IgAN model at least because the treatment with shNLRP3 could not entirely prevent or improve the IgAN mice. This proposition should be warranted for further investigation.

## Methods

### Preparation of IgA antibody and PnC

T15 hybridoma-derived, purified IgA anti-phosphorylcholine antibody and PnC were prepared as described previously^7^,^20^,^21^,^42^,^43^.

### Animals

For IgAN induction, 8-week-old, female, NLRP3 KO mice (Center for Molecular and Clinical Immunology, Chang-Gung University, Tao-Yuan, Taiwan) and C57BL/6 mice (WT) (National Laboratory Animal Center, Taipei, Taiwan) were used. Briefly, IgAN was induced by 36 daily injections of purified IgA anti-phosphorylcholine antibodies and PnC into NLRP3 KO mice and WT mice as described previously^7^,^20^,^21^,^42^,^43^. For OVA-specific T cell proliferation assay, 8-week-old, female OT-II mice (provided by Dr. C. Lowell, University of California, San Francisco, CA) were used as described previously^47^. All animal experiments were performed after approval by the Institutional Animal Care and Use Committee of the National Defense Medical Center, Taiwan and were consistent with the NIH G*uide for the Care and Use of Laboratory Animals.*

### Cell cultures

Murine J774A.1 macrophages (TIB-67™), murine M-1 renal TECs (CRL-2038™), and 293 T cells were obtained from the American Type Culture Collection. Mouse peritoneal macrophages^48^, BMDCs^47^, and glomerular PMCs^49^ were prepared from NLRP3 KO and WT mice as described previously. All cells were cultured at 37 °C in a 5% CO_2_ incubator.

### Clinical and pathological assessment

Urine samples were collected in metabolic cages and albuminuria determined by the urine albumin/Cr ratio in samples taken at 0, 7, 14, 28, and 36 days as described previously^50^. The mice were sacrificed at day 14 and day 36 after IgAN induction, and renal cortical tissue and blood samples taken, respectively. Serum levels of BUN and Cr were measured using BUN kits or Cr kits (both from Fuji Dry-Chem Slide, Fuji Film Medical). Renal tissues were fixed in 10% formalin and paraffin-embedded, then 4 um thick sections were cut and stained with hematoxylin and eosin. Scoring of the severity of renal histopathology were performed as described previously^20^. The percentage of glomeruli showing glomerular proliferation, glomerular sclerosis, or periglomerular mononuclear leukocyte infiltration was determined by counting in 50 randomly sampled glomeruli by light microscopy at a magnification of x400.

### Immunohistochemical analysis

Methyl Carnoy’s solution-fixed, paraffin-embedded renal sections were used for the detection of CD3^+^ cells (pan-T cells, Dako) and F4/80^+^ cells (macrophages; Serotec), while frozen sections of renal tissues were used for the detection of CD11c^+^ cells (DCs, BD Biosciences). The number of CD3^+^, F4/80^+^, and CD11c^+^ cells were counted at a magnification of x400 in 50 randomly selected glomeruli or in 20 randomly selected fields of the tubulointerstitial compartment in the renal cortex using Pax-it quantitative image analysis software (Pax-it; Paxcam) as described previously^51^.

### Western blot analysis

Protein lysates from renal cortical tissues and cultured cells, and supernatants from cultured cells were run on 10% or 15% SDS–PAGE gels. Mouse against NLRP3 (AdipoGen), rabbit against IL-1β, IL-18, caspase-1 or goat anti-β-actin antibodies (all from Santa Cruz Biotechnology) were used as primary antibodies and horseradish peroxidase-conjugated rabbit anti-goat, goat anti-rabbit or goat anti-mouse IgG antibodies as secondary antibodies (all from Santa Cruz Biotechnology). The membrane-bound antibody detected was incubated Enhanced Reagent Plus (PerkinElmer Life Sciences) and PVDF membranes were scanned with an UVP BioSpectrum Imaging Systems (Financial HealthCare) as described previously^52^.

### Enzyme-linked immunosorbent assays (ELISA) and enzyme activity assay

IL-1β, TNF-α, IL-17A, IL-4, and IFN-γ levels in serum or supernatants of cultured cells were measured using ELISA kits (all from R&D Systems), while caspase-1 activity in lysates was measured using a caspase-1 fluorometric kit (R&D Systems) according to the manufacturer’s instructions.

### Real-time PCR

RNA was extracted using TriZOL reagent (Invitrogen) and cDNA prepared as described previously^50^. The primers used were: for mouse caspase-11, forward 5-GCCACTTGCCAGGTCTACGAG-3 and reverse 5-AGGCCTGCACAATGATGACTTT-3; for mouse GAPDH, forward 5-TCCGCC CCTTCTGCCGATG-3 and reverse 5-CACGGAAGGCCATGCCAGTGA-3. Mitochondrial DNA in the cytosol was extracted as described previously^27^. The primers used were: for mouse cytochrome oxidase I, forward 5-GCCCCAGATATAGCATTCCC-3, and reverse 5-GTTCATCCTGTTCCC-3, and for mouse 18S, forward 5-TAGAGGGACAAGTGGCGTTC-3, and reverse 5-CGCTGAGCCAGTCAGTGT-3. Real-time PCR was performed on an ABI Prism 7700 Sequence Detection System (Applied Biosystems) using SYBR Green mix (Thermo Scientific).

### Flow cytometry

Isolated splenocytes were stained to measure activation of T cells using allophycocyanin (APC)-conjugated anti-CD4 (RM4-5) or CD8 (53-6.7) antibodies, phycoerythrin (PE)-conjugated anti-CD62 antibodies (MEL-14), or fluorescein isothiocyanate (FITC)-conjugated anti-CD44 antibodies (IM7) (all from BD Biosciences). The cells were stained for Treg cells using the Mouse Regulatory T cell Staining Kit (eBioscience) according to the manufacturer’s instructions. Maturation of BMDCs was determined by the upregulation of CD11c^+^ and costimulatory molecules expression, the cells were stained with PE-conjugated anti-CD11c antibody, then with FITC-conjugated anti-CD40 or anti-CD86 antibodies (all from BD Biosciences), respectively, as described previously^47^. To measure percentage of CD4^+^ IL-17A^+^ T cells, intracellular cytokine staining was performed and the cells were stained with APC-conjugated anti-CD4 antibody, then with PE-conjugated anti-IL-17 antibody (BD Biosciences) as described previously^53^. Cells were analyzed by a FACSCalibur (BD Biosciences) as described previously^20^.

### ROS detection

Production of total ROS in J774A.1 macrophages was determined by measuring the intensity of fluorescence of 2′, 7′-dichlorofluorescein, the oxidation product of 2′, 7′-dichlorofluorescein diacetate (Molecular Probes), while mt ROS production was assessed by measuring the fluorescence of MitoSOX (Invitrogen) as described previously^33^.

### Stable expression of shRNAs

Lentivirus transduction particles carrying shNLRP3 or caspase-11 (National RNAi Core Facility, Academia Sinica, Taipei, Taiwan) in 293 T cells were constructed. J774A.1 macrophages or TECs were then infected with lentivirus-bearing specific shRNAs of NLRP3 or caspase-11, respectively, and incubated with puromycin (Invitrogen) to select stably-infected cells for further experiments as described previously^54^.

### OVA-specific T cell proliferation assay

BMDCs from NLRP3 KO or WT mice were incubated with IgA ICs for 24 h, then incubated 18 h with OVA_323–339_ peptide (1 μg/ml) (Genomics) and CD4^+^ T cells derived from OT-II mice, and T cell proliferation was measured by [^3^H] thymidine incorporation as described previously^47^.

### Ultrasound-mediated, kidney-targeting shNLRP3 gene transfer into mice

Plasmids of shNLRP3-luciferase was generated by conjugating shNLPR3 (Santa Cruz) with luciferase as described previously^55^. The plasmids in 200 μl of saline was mixed with 200 μl of SonoVue® microbubbles (Bracco), and the mixture injected into mice via the tail vein, followed by application of transcutaneous ultrasound to the back at the level of the kidney using a Sonopuls 590 at 1 MHz (Ernaf-Nonius) as described previously^50^. To determine the time-course of shNLRP3 expression, and the interval at which shNLRP3 plasmids were injected into WT mice, the mice received the shNLRP3 plasmids (200 μg) and monitored for the presence of luciferase using an real-time imaging system IVIS 100 series (Xenogen Corp.) on days 0, 1, 2, 3, and 7 after the delivery. Control mice were injected with shSC plasmids. To evaluate the renoprotective effect of shNLRP3 on IgAN mice, the plasmids were given every 3 days starting one day before induction of IgAN, while, to evaluate the therapeutic effect on diseased mice, the treatment was given every 3 days starting seven days after induction of the disease.

### Statistical analyses

Results were presented as the mean ± SEM. Comparisons between two groups were performed using Mann-Whitney U test. The significance of differences in [1] urinary albumin/Cr levels and [2] the level of bioluminescence activity of injected shNLRP3 *in vivo* assessed by IVIS was examined using Kruskal-Wallis test. For the *in vitro* experiments, data analysis involved Kruskal-Wallis test. A value of *p* < 0.05 was considered statistically significant.

## Additional Information

**How to cite this article**: Tsai, Y.-L. *et al*. NLRP3 inflammasome: Pathogenic role and potential therapeutic target for IgA nephropathy. *Sci. Rep.* **7**, 41123; doi: 10.1038/srep41123 (2017).

**Publisher's note:** Springer Nature remains neutral with regard to jurisdictional claims in published maps and institutional affiliations.

## Figures and Tables

**Table 1:** Serum levels of proinflammatory cytokines.

Conc. (pg/ml)	Day 14				Day 36			
	WT + saline	NLRP3 KO + saline	WT + IgAN	NLRP3 KO + IgAN	WT + saline	NLRP3 KO + saline	WT + IgAN	NLRP3 KO + IgAN
IL-1β	103.3 ± 7.2	110.6 ± 4.3	154.4 ± 5.2^‡‡‡^	106.7 ± 8.8^***^	107.4 ± 2.4	106.8 ± 3.0	183.5 ± 3.8^‡‡‡^	113.1 ± 9.5^***^
TNF-α	121.0 ± 5.8	118.7 ± 4.8	196.8 ± 26.1^‡‡^	163.6 ± 14.1^‡^	116.0 ± 13.4	120.0 ± 2.7	385.0 ± 80.5^‡‡‡^	193.5 ± 14.7^‡‡*^
IL-17A	6.2 ± 0.8	6.2 ± 0.7	6.1 ± 0.5	6.2 ± 0.9	6.0 ± 0.7	6.0 ± 0.5	18.9 ± 1.7^‡‡^	5.0 ± 0.2^***^
IL-4	3.3 ± 0.2	3.1 ± 0.3	3.2 ± 0.3	3.2 ± 0.2	3.2 ± 0.3	3.1 ± 0.5	3.3 ± 0.4	6.7 ± 0.6^‡‡**^
IFN-γ	5.0 ± 0.8	5.3 ± 0.7	8.6 ± 1.6	6.3 ± 0.7	5.1 ± 0.4	6.3 ± 0.1	16.8 ± 5.1^‡^	5.2 ± 1.8

ELISA with serum samples taken from the mice on days 14 and 36 after the induction of IgAN (WT + IgAN mice; NLRP3 KO + IgAN mice) and respective saline controls (WT + saline mice; NLRP3 KO + saline mice). The data are expressed as the mean ± SEM for 7 mice per group. *p < 0.05, **p < 0.01, ***p < 0.005 compared to wild type mice. ^‡^*p* < 0.05, ^‡‡^*p* < 0.01, ^‡‡‡^*p* < 0.005 compared to saline control mice. WT, wild type.
